# A community-based educational initiative to support awareness of relative energy deficiency in sport among adolescent athletes

**DOI:** 10.3389/fpubh.2026.1868398

**Published:** 2026-08-04

**Authors:** Nikola Cizlerová, Tomáš Hlinský

**Affiliations:** Faculty of Sports Studies, Masaryk University, Brno, Czechia

**Keywords:** adolescent athletes, community education, low energy availability, prevention, sports nutrition

## Abstract

Relative Energy Deficiency in Sport (REDs) is a syndrome caused by chronic low energy availability (LEA) and can disrupt hormonal, metabolic and psychological functioning. Adolescents are particularly vulnerable because they are still growing, have high energy requirements and are susceptible to sociocultural pressures. Despite increasing scientific attention, awareness and prevention of REDs remain insufficient in everyday sport settings. This community case study describes the Energy for Long-Term Health initiative, implemented in 2025–2026 in the Czech Republic across six sports-focused secondary schools (≈300 students, aged 15–19 years). Each school received an interactive workshop (60–120 min) structured into four blocks: (1) explanation of energy availability and its practical implications; (2) consequences of LEA for health and performance; (3) recognition of warning signs and risk situations; and (4) preventive strategies and the role of the support team. Secondary target groups included coaches, teachers and parents, who were engaged through reusable visual materials and online dissemination via the online platform. Key lessons were that adolescent REDs education requires age-appropriate language, opportunities for anonymous questions and careful facilitation of sensitive topics. Combining face-to-face workshops with online resources extended reach and reinforced key messages. Although no formal evaluation of effectiveness was conducted, high attendance and positive informal feedback suggest that university-supported community initiatives can enhance awareness of REDs among young athletes and their support networks. Future applications should incorporate formal needs assessments, systematic evaluation, and validated measures of knowledge and behaviour change.

## 1 Introduction

Competitive sport participation during childhood and adolescence is associated with increasing training loads, dietary control and a quest for optimised body composition (1). Energy intake is often framed mainly as a means to improve athletic performance, yet its essential roles in growth, endocrine regulation and long-term health are frequently overlooked (2, 3). Prolonged mismatch between energy intake and energy expenditure leads to LEA, defined as the dietary energy remaining for basic physiological functions after accounting for exercise expenditure relative to fat-free mass (4). Chronic LEA underpins Relative Energy Deficiency in Sport (REDs), a syndrome that can impair menstrual and reproductive function, bone health, cardiovascular and immune systems, psychological wellbeing and performance (5, 6).

Adolescence represents a period of heightened vulnerability to LEA due to accelerated growth, hormonal maturation, and sociocultural pressures related to performance and body image (7, 8). Despite substantial scientific knowledge on the physiological and psychological consequences of REDs in both females (9) and males (10), prevention and early recognition in school and club settings remain limited (11). Warning signs such as persistent fatigue, frequent illness, irregular menstrual cycles, or impaired concentration may be normalised as typical consequences of training (3, 12). Community-based health education initiatives targeting adolescents and their support networks are therefore essential but scarcely reported, especially in Central and Eastern Europe. This community case study describes the development, implementation and lessons learned from the community project, a university-supported initiative aimed at improving REDs awareness among adolescent athletes in the Czech Republic.

## 2 Context

Supported by the ComMUNity Fund of Masaryk University, which aims to support initiatives addressing specific societal needs in the local environment, the project named “Energy for Long-Term Health” was carried out throughout the year 2025 in the Czech Republic. The project emerged as a response to repeatedly observed low awareness of energy availability among young athletes and their support networks, as described in the literature (11, 13, 14). The literature also suggested that primary prevention targeting all individuals involved in the process of athletes’ training and development, through educational workshops, can affect body image and reduce disordered eating, eating-disorder risk factors, and/or LEA (15–19). A few observational studies on the publication of student theses were conducted in recent years using the REDs Screening Tool (RST) questionnaire, published by Foley Davelaar et al. (41), and EA evaluation, based on dietary and training reports, among the youth athletic population at our faculty (20–24). In this case, a gap was identified between the available scientific knowledge on REDs and the education in their application in the everyday lives of adolescent athletes. The project thus represented the university’s community-based response to the specific educational needs of regional sports-oriented secondary schools.

The primary target group of this project consisted of students from sports-focused secondary schools. Six sports-focused secondary schools and grammar schools that include sport-oriented classes were purposively selected, also based on the existing collaborations with the Faculty of Sports Studies. At these schools, the topic of REDs prevention was considered particularly relevant due to the regular demands of training and performance expectations. Students were aged 15–19 years (mean 16.8 ± 1.1); approximately 55% were male and 45% female. The most common sports were football (35%), athletics (20%), swimming (15%), and gymnastics (10%), with the remaining students participating in sports such as tennis, basketball, or rowing. The secondary target group included parents, coaches, and educators. Coaches and physical education teachers were invited to attend the sessions, and in three schools, two to four coaches were present. Parents were not present at the workshops, but were reached through school newsletters and online materials.

Participation in all workshops was voluntary and coordinated through the participating schools. School leaders approved the seminars as part of their existing prevention and educational programmes, and parents were notified through the schools with the option to withdraw their children. Students aged 15–19 could decide for themselves whether to attend, because no sensitive personal data was collected. According to the Czech Ministry of Education’s Ethical Framework and related guidelines, written parental consent is not mandatory for social research with adolescents older than 14. However, the school’s approval and students’ voluntary assent are emphasised. By working through the school administrations, informing parents, and ensuring that participation was entirely voluntary, the project operated within these national ethical expectations.

Each workshop session was attended by approximately 30–60 students, which allowed for an interactive format while reaching a broader group of young athletes. A total of six sessions were delivered, reaching approximately 300 students. All sessions followed a unified structure but were flexibly adapted to the specific school environment.

This combination of institutional collaboration, repeated implementation, and engagement with different types of sports-oriented schools supports the characterisation of the project as a locally embedded yet potentially transferable community health education initiative. Although the initiative was not designed as a research intervention, it was rather a community service aimed at improving health literacy in sports nutrition.

## 3 Key programmatic elements

Each workshop lasted 60–120 min, depending on the school’s organisational constraints. The workshop’s structure consisted of four blocks, each lasting 20–30 min (Table 1). Duration was flexibly adjusted to each school’s available time. All the content and educational materials were in Czech.

**Table 1:** Workshop structure.

Block	Key concepts and activities	Materials	Intended learning outcomes
Understanding energy availability	Short lecture on energy balance and EA; group brainstorm on daily energy demands; interactive calculation of EA for a typical day.	Slide deck; infographic on EA; worksheet with example calculations.	Students can define EA and recognise its role in growth, hormonal regulation and performance.
Consequences of low energy availability/REDs	Presentation of physiological and psychological consequences of LEA and REDs, using age-appropriate language; discussion of case stories; myth-busting quiz.	Visual infographic of REDs symptoms (bone, endocrine, immune, psychological); printed myth-vs.-fact cards.	Students identify at least three potential consequences of REDs and dispel common misconceptions.
Identifying warning signs and risky situations	Group exercise to list warning signs; anonymous poll via Slido to submit personal observations; discussion of risk factors (e.g., sudden training increases, restrictive dieting).	Slido anonymous polling; handout summarising warning signs; example scenario script.	Students recognise early signs of LEA and understand contexts that increase risk in their sports.
Prevention and support team roles	Discussion on balanced nutrition, appropriate training progression, importance of rest; mapping of the support network (coaches, parents, nutritionists); role-playing conversations seeking help.	Workshop handbook; referral guide to local dietitians; contact cards; Naplno website link.	Students know at least two preventative strategies and where to seek help; support persons understand their role in prevention.

The project was implemented by student facilitators from the Faculty of Sports Studies under the professional supervision of an academic staff member. These facilitators had already completed a bachelor’s degree in Regeneration and Nutrition in Sport and were continuing in follow-up Master’s studies. They were guided by the principal investigator/project lead and delivered the workshops in accordance with a shared structure and content framework. The programme content was based on current international consensus recommendations (5) and review studies on LEA (2, 12).

The main learning goals of the programme were to increase awareness of REDs and energy availability among adolescent athletes, provide education aimed at primary prevention, improve recognition of warning signs and symptoms, and offer clear signposting to appropriate sources of further help and professional support when concerns arose (14, 25).

There were key elements to the community project:

### 3.1 Interactive workshops at secondary schools

The workshops were structured into several thematic blocks:

- Explanation of the concept of energy availability;
- effects of REDs on health and performance;
- recognition of warning signs;
- prevention and the role of the support team (coach, parent, health professional).

The programme included model situations based on typical scenarios from sports practice. For example, the case of a young athlete who increased training load, simultaneously restricted energy intake to “improve form,” and subsequently experienced increased fatigue, more frequent illness, and decreased performance was discussed. Participants were asked to identify potential risk factors and propose appropriate preventive measures.

Sensitive topics, such as body weight, body composition, and the menstrual cycle, were addressed using respectful, non-judgmental language. Instead of individualised questioning, general scenarios and an anonymous collection of questions via the digital platform Slido were used. This tool allowed students to ask questions anonymously, which proved to be an important factor in discussing topics that may be associated with shame or stigma. The safety of the discussion was further supported by clearly communicated rules of respect, voluntary sharing of personal experience, and the option to withdraw from the discussion at any time. After the anonymous part, a voluntary open discussion followed, providing space for deeper reflection. This two-phase model (anonymous input, voluntary interaction) was a practical strategy for addressing sensitive REDs-related topics among adolescents. This approach allowed the theoretical concept of energy availability to be translated into a concrete, student-understandable framework.

Special attention was paid to widespread myths about nutrition, body weight, and the “ideal” athletic body. The discussion format enabled open communication and reduced the stigmatisation of the topic.

### 3.2 Visual educational materials and infographics

Clear materials summarising key information on energy availability, risk symptoms, and preventive recommendations were created. These materials were designed for long-term use in schools and sports clubs.

Educational materials were also developed in cooperation with the Naplno project, which promotes a healthy lifestyle among young athletes. This collaboration not only enriched the materials’ content but also extended their reach into the online environment. Key messages were adapted for digital sharing, primarily through the Naplno project’s website and Instagram channels, enabling outreach to a broader target group beyond the direct workshop participants (26, 27).

### 3.3 Popularisation activities at the university

The project was also presented during university events aimed at collaboration with secondary schools, thereby enhancing the transfer of expert knowledge to the wider community. At the same time, information about the project and related popularisation texts was shared through faculty news channels, further increasing the project’s impact beyond direct workshop participants. The programme was designed as preventive, not diagnostic. Participants were not individually assessed, and no health data were collected. The combination of in-person education, reusable materials, and online dissemination represented an important implementation feature intended to support continued visibility of the topic beyond single workshop sessions.

From an implementation perspective, the programme model consisted of a standardised workshop structure, trained facilitators with a background in sport nutrition, purposive selection of sport-focused school settings, and reinforcement of key messages through reusable visual materials and online dissemination.

## 4 Discussion

The presented community project is a local initiative aimed at improving health literacy regarding EA and REDs among adolescent athletes. In the context of sports-oriented secondary schools in the Czech Republic, this case study provides a practical example of how university-supported education can be integrated into school-based sport environments to raise awareness of REDs and energy availability. However, because no formal feasibility, acceptability, or effectiveness evaluation was conducted, the present initiative should not be interpreted as evidence of impact. Instead, it offers implementation experience and practical lessons that may inform future REDs-related educational and primary prevention initiatives in similar school and sport settings.

### 4.1 Practical implications and lessons learned

A major strength of the initiative was the combination of direct instruction with reusable supporting materials, including infographics and concise recommendations that could continue to serve students, coaches, parents, and educators after the workshops ended. Complex communication is particularly relevant because REDs cannot be understood through a single isolated sign or symptom; both prevention and early recognition require a broader understanding of energy availability in relation to training load, nutrition, recovery, psychological factors, and, where appropriate, clinical evaluation (5).

An important practical lesson was that REDs-related education for adolescents should be framed not only in terms of performance but also in relation to long-term health, development, and sustainable participation in sport. This perspective is especially relevant during adolescence, when chronic energy deficiency may negatively affect bone health, endocrine regulation, growth, and overall wellbeing during a life stage critical for future health outcomes (2, 5, 28–32). More importantly, approximately 90% of adult peak bone mass is achieved by the end of the second decade of life, with roughly 26% accrued during the 2 years around peak height velocity. Energy intake, weight-bearing exercise, calcium/vitamin D intake and sex-steroid hormones interact to determine bone accrual. Chronic LEA during this period may impair menstrual function, reduce bone mineral density and have long-lasting consequences. Therefore, when an adolescent exhibits weight loss, performance decline, mood changes, frequent illness or fractures, or menstrual irregularities, LEA can be suspected (33, 34).

Beyond physiological factors, psychological and social pressures are increasingly recognised in REDs. A scoping review reported that the social fixation on a “perfect” body in sports leads many athletes to pursue muscular or lean physiques, often resorting to diets, which can result in weight fluctuations, body dissatisfaction, and disordered eating (35–37). These behaviours lower EA and are a key step on the path to REDs (38). Negative body image and the drive to be thinner or more muscular can trigger restrictive eating or excessive exercise, even when athletes have adequate nutritional knowledge. Constant comparison through traditional and digital media reinforces body-related ideals and performance pressure. Social media is highlighted as an additional risk factor for body dissatisfaction, unhealthy eating behaviours and energy imbalance (39). Research also shows that comments from coaches or other key figures about weight or body shape can encourage restrictive practices and risk of injury, underscoring the need for sensitive communication (40).

The project also highlighted the importance of age-appropriate and context-sensitive communication. Technical or overly clinical language alone is unlikely to engage adolescent participants effectively. In contrast, examples drawn from school and sport life, such as fatigue, recurrent illness, pressure to change body composition, or uncertainty about “healthy eating” in sport, provided a more accessible framework for discussing EA in a meaningful way (11).

Another important lesson concerned the broader support system around young athletes. Although the workshops primarily targeted students, the implementation confirmed the importance of parents, coaches, and teachers in shaping norms related to performance, recovery, nutrition, and body image. If adults perceive warning signs of LEA as normal or desirable, preventive messages targeted only to adolescent athletes are unlikely to be effective (3, 5). For this reason, throughout the project, materials accessible beyond the immediate workshop setting were developed, along with references to the project partners’ websites and materials to inform participants further.

A further practical insight was the need to create safe conditions for discussing sensitive topics. REDs-related education often touches on issues associated with embarrassment, stigma, or fear of judgement, including body weight, body composition, restrictive eating, menstrual health, and pressure from coaches or peers. In this context, the use of anonymous question collection via Slido proved a valuable implementation strategy, enabling participation from students who might otherwise have remained silent (11).

The two-phase format, consisting of anonymous questions followed by voluntary open discussion, also helped balance participation with respect for personal boundaries. The anonymous component allowed students to ask about sensitive issues without self-exposure, while the voluntary discussion created space for clarification and deeper reflection. From a transferability perspective, this element appears easily adaptable to other school and club settings and may be especially useful in health education contexts where shame or stigma can reduce engagement.

The project further suggested that a hybrid educational model may be particularly useful in this area. In-person workshops allowed for explanation, dialogue, and contextualisation, while online dissemination via the Naplno project’s website and Instagram channels reinforced key messages beyond the single educational session (26, 27). This was especially relevant for an adolescent population whose everyday information environment is strongly shaped by digital communication. Online dissemination also extended the initiative’s reach to secondary target groups, including parents, coaches, and teachers, who may not have participated directly but still play an important role in shaping the athlete’s environment.

From a broader public health perspective, the initiative illustrates a potentially valuable role for universities as intermediaries between scientific knowledge and community practice. In this case, the university provided expert supervision, evidence-informed content, and trained facilitators to deliver a preventive educational programme in response to locally identified needs. Furthermore, based on the 2023 IOC consensus statement, universities can provide detection tools (e.g., REDs CAT2, RST questionnaire, LEAF-Q/LEAM-Q) and a multidisciplinary management approach involving medical, nutritional, and psychological expertise (5).

## 5 Limitations

The experiences from this initiative should be interpreted in light of several limitations. First, this article describes a single community-based case rather than a controlled intervention. Case studies are valued for the richness of context, but they are frequently criticised for lacking scientific rigour and offering little basis for generalising results to broader populations. We did not collect systematic pre- and post-intervention data on knowledge, attitudes, behaviour or health outcomes, so it is impossible to quantify acceptability, feasibility or effectiveness. Because the programme was planned and delivered by the authors, there is also a risk of researcher bias. Case study findings often rely on the researcher’s subjective interpretation and proximity to participants. As a result, the project does not permit conclusions regarding effectiveness in reducing REDs risk or changing health outcomes.

Second, no clinical, anthropometric or screening data were gathered. This was a deliberate choice to maintain the preventative, non-diagnostic character of the workshops and to foster psychological safety. However, it precludes any assessment of participants’ baseline risk factors or subsequent changes, and it limits the ability to evaluate whether the educational content matched individual needs.

Third, the initiative was implemented in a specific regional and institutional context with support from receptive school leadership, access to trained facilitators and established university–community partnerships. These contextual facilitators may limit transferability. The generalisability of a single case is debated and is typically based on “analytic generalisation” to theory rather than statistical inference. Factors such as school culture, scheduling constraints, and resource availability may influence the feasibility of replication in other settings.

Fourth, although the workshops followed a standard structure under professional supervision, there was inevitably some variation in delivery style. Without systematic qualitative feedback from students, teachers or coaches, we cannot assess how this variability influenced participant engagement or learning. The absence of in-depth qualitative process evaluation – a recognised strength of well-designed case studies – is a missed opportunity; as noted in methodological critiques, many case studies omit clear descriptions of their design, thereby hindering assessment of credibility.

Finally, as a general preventive programme, the intervention could not address individual clinical situations requiring multidisciplinary assessment or treatment (5). Recognising warning signs and making appropriate referrals to healthcare professionals were emphasised, but individual diagnosis and management of REDs were beyond the project’s scope.

## 6 Future directions

Future implementations of this model should incorporate ethically approved evaluation strategies. Anonymous pre- and post-assessments using validated questionnaires (e.g., LEAF-Q/LEAM-Q for low energy availability or NSKQ for sport nutrition knowledge tests) could measure changes in awareness and self-efficacy, while simple surveys could capture acceptability and perceived relevance. Qualitative feedback from students, teachers and coaches—via focus groups, reflective diaries or open-ended questionnaires—would provide nuanced insights into which components are most engaging and what adaptations are needed to different sports or school environments.

Greater systematic involvement of secondary target groups (parents, coaches and educators) may enhance long-term impact. This could include parent workshops, coach training sessions or integrating REDs topics into teacher professional development. Partnerships with school boards and sporting federations could facilitate broader adoption and sustainability.

To address the limitation of transferability, future projects should pilot the intervention in diverse educational and cultural contexts and compare experiences across multiple cases. As methodological literature notes, findings from case studies are more generalisable when multiple cases reveal similar patterns. Comparative case study designs or mixed-methods evaluations would help distinguish context-specific factors from core elements of successful REDs education.

Additionally, collaboration with researchers specialising in adolescent development, psychology and public health could help integrate frameworks that address peak bone mass accrual, puberty, psychological vulnerability and social media influence. Combining community education with formal needs assessments and rigorous evaluation would move beyond illustrative implementation toward evidence-based practice.

Overall, this community case study does not demonstrate the intervention’s effectiveness. Still, it offers a practical example of how REDs-related health education can be implemented in collaboration with sports-oriented secondary schools. The lessons learned may be relevant to similar school- and community-based efforts seeking to improve awareness of EA, reduce stigma around sensitive topics, and promote healthier, more sustainable participation in sport among adolescents.

## 7 Conclusion

Relative Energy Deficiency in Sport represents a significant yet often overlooked risk to the health of adolescent athletes. The community project “Energy for Long-Term Health” demonstrates that a university-supported community education initiative can serve as a practical model for increasing awareness of EA and REDs in adolescent sport settings. Strengthening health literacy about EA is a key step toward protecting young athletes’ future health.

## Data Availability

The original contributions presented in the study are included in the article/Supplementary material, further inquiries can be directed to the corresponding author/s.
