# Supplementary material for: A community-based educational initiative to support awareness of relative energy deficiency in sport among adolescent athletes
Source: Front Public Health. 2026 Aug 4;14:1868398. doi: 10.3389/fpubh.2026.1868398 (PMC13481860; doi:10.3389/fpubh.2026.1868398)
Supplement: Supplementary file 1 [file Data_Sheet_1.PDF]

Energetický příjem

Sportovní trénink a regenerace jsou energeticky náročné a pro zlepšování výkonů a udržení se ve zdraví je třeba vydanou energii dostatečně doplňovat. Často ale nestačí spoléhat se pouze na pocit hladu.

1. Všechny živiny jsou důležité

Sacharidy jsou primárním zdrojem energie a důležité pro správnou funkci imunitního systému. Bílkoviny jsou důležité pro správnou regeneraci svalů, ale i ostatních tkání a správnou funkci enzymů. Tuky jsou nezbytné pro vstřebávání některých vitamínů a hormonální zdraví.

2. Důraz na dostatečný příjem sacharidů

Nízká dostupnost sacharidů Ovlivňuje schopnost svalů použít a obnovit glykogen, což limituje výkon při aktivitě vysoké intenzity a může vést k symptomům REDs syndromu.

3. Sportovní výživa ≠ pouze zdravá výživa

Výživa sportovce se liší od výživy běžné populace, proto se s lidmi v tvém okolí neporovnávej. Sport klade vysoké nároky na tvoje tělo, proto je ho třeba i dostatečně vyživovat.

SLOŽENÍ STRAVY

Konkrétní skladba a množství bude u každého individuální, ale obecně se můžeš řídit tímto rozložením.

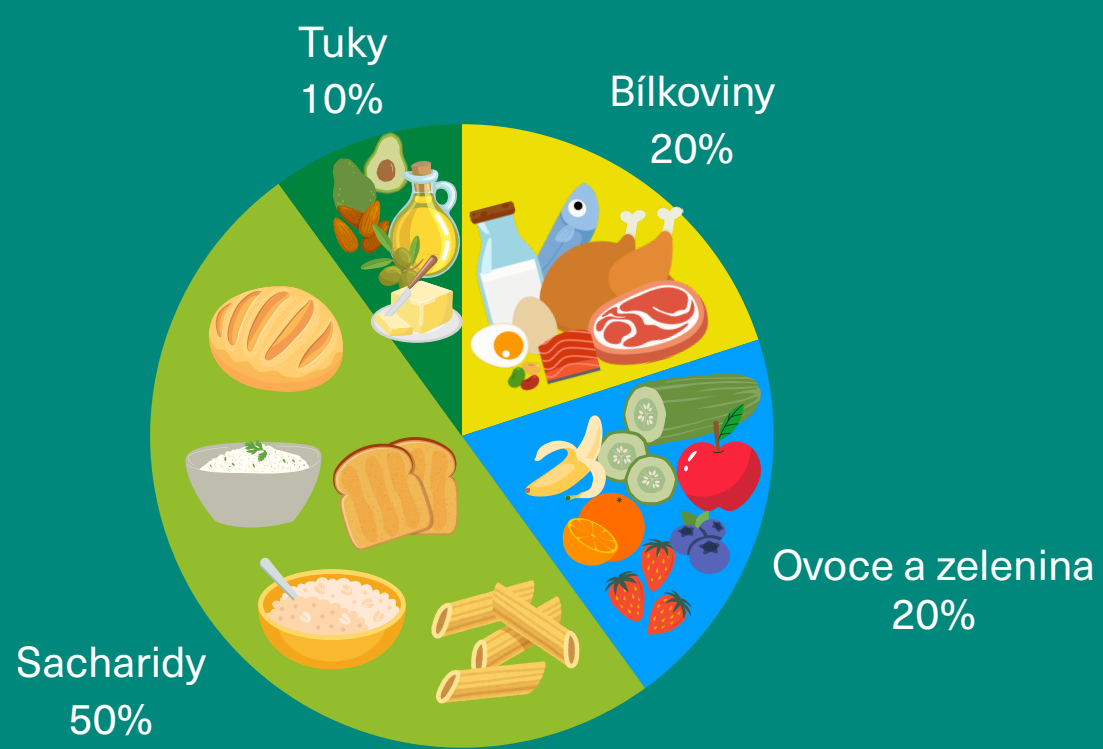

REDs

Příručka pro sportovce

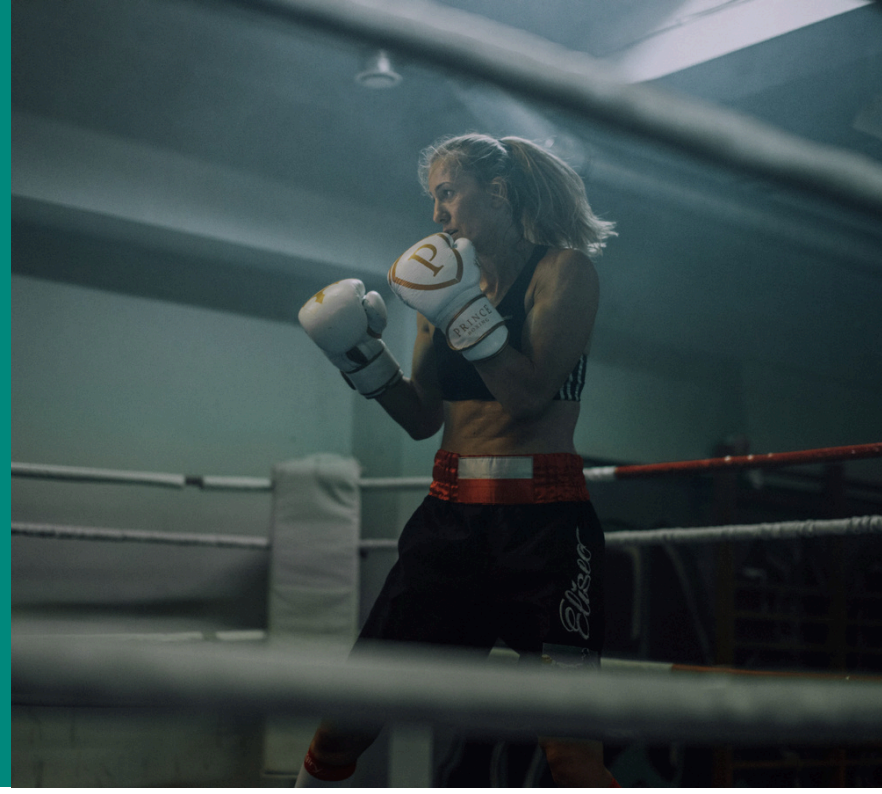

Co je to REDs?

Relativní energetická nedostatečnost ve sportu znázorňuje dopady problematické nízké energetické dostupnosti na zdraví i výkonnost. Jako jsou například dysfunkce menstruačního cyklu, narušené zdraví kostí nebo snížená výkonnost.

Nízká energetická dostupnost

Příčinou může být zvýšení tréninkové zátěže, bez adekvátního zvýšení energetického příjmu, narušené stravovací zvyklosti nebo poruchy příjmu potravy nebo neúmyslný nedostatečný energetický příjem.

Menstruační cyklus

Nepravidelná nebo chybějící menstruace je varovný signál! Sleduj svůj cyklus a při jakékoli problematice zpozorni. Více jak 60 % vytrvalostních sportovkyň trpí menstruačními disfunkcemi. Chybějící nebo problémová menstruace ale není normální ani správná!
Pozor, hormonální antikoncepce může projevy signalizující problém překrývat.

Trpělivost

Během puberty tělo prochází spoustou fyzických i psychických změn, které mohou ovlivnit, jak se sportovec cítí a jaké podává výkony. Upřednostňuj dlouhodobé zdraví nad krátkodobými výkonnostními cíli.

MUNI FAKULTA SPORTOVNÍCH STUDIÍ

@sport_muni

www.fsps.muni.cz

NAPLNO

@naplnocz

www.projektnaplno.com
